# Effects of a Novel *Hippophae rhamnoides* L. Seed Lipid Extract Formulation Obtained via Supercritical Carbon Dioxide Extraction on Reducing Residual Cardiovascular Risk in High-Risk Patients: A Pilot Study

**DOI:** 10.3390/jcdd12120478

**Published:** 2025-12-04

**Authors:** Kristaps Erglis, Baiba Kokina, Sanda Jegere, Iveta Mintale, Eriks Jakobsons, Vadims Bartkevics, Martins Erglis, Ralfs Zuzans, Inga Narbute, Karlis Trusinskis, Andrejs Erglis

**Affiliations:** 1 AS “SISTEMU INOVACIJAS” (sibiotech), Saulrieta Krastmala 55, LV-1063 Riga, Latvia; kristaps@sibiotech.com (K.E.); eriks.jakobsons@stradini.lv (E.J.); 2 Institute of Cardiology and Regenerative Medicine, University of Latvia, Jelgavas Street 3, LV-1004 Riga, Latvia; inga.narbute@stradini.lv; 3 Faculty of Medicine and Life Sciences, University of Latvia, Jelgavas Street 3, LV-1004 Riga, Latvia; sanda.jegere@stradini.lv (S.J.); iveta.mintale@stradini.lv (I.M.); martins.erglis@stradini.lv (M.E.); karlis.trusinskis@gmail.com (K.T.); 4 Faculty of Residency, Riga Stradins University, Dzirciema Street 16, LV-1007 Riga, Latvia; baiba.kokina@gmail.com; 5 Latvian Centre of Cardiology, Pauls Stradins Clinical University Hospital, Pilsonu Street 13, LV-1002 Riga, Latvia; ralfs.zuzans@stradini.lv; 6 Institute of Food Safety, Animal Health and Environment “BIOR”, Lejupes Street 3, LV-1076 Riga, Latvia; vadims.bartkevics@bior.lv; 7 Centre of Cardiac Surgery, Pauls Stradins Clinical University Hospital, Pilsonu Street 13, LV-1002 Riga, Latvia

**Keywords:** sea buckthorn, macronutrients, unsaturated fatty acids, cardiovascular risk, dyslipidemia, low-density lipoprotein cholesterol, inflammation

## Abstract

Unsaturated fatty acids have the potential to reduce residual cardiovascular risk. Sea buckthorn (*Hippophae rhamnoides* L.) contains several valuable bioactive substances, including lipids with a balanced fatty acid composition. The aim of this study was to evaluate the effects of sea buckthorn seed lipid extract (SBS-LE) on residual cardiovascular risk in high-risk patients. In this pilot study, 86 patients with chronic coronary syndrome receiving statin (atorvastatin or rosuvastatin) and/or ezetimibe were enrolled. SBS-LE capsules (1000 mg, twice daily) were prescribed in addition to standard medical therapy, with each capsule containing 300 mg of omega-3 alpha-linolenic acid, 370 mg of omega-6 linoleic acid, 170 mg of omega-9 oleic acid and 7 mg of the plant sterol beta-sitosterol. For this clinical trial, SBS-LE was produced via supercritical fluid extraction with carbon dioxide. Clinical effects and impacts on laboratory test results were evaluated at baseline and after three months. Additionally, lipidomics testing was performed to confirm the bioavailability of the formulation. Significant reductions in systolic blood pressure by 2.9 mmHg (2.1%, *p* = 0.012), LDL-C by 0.3 mmol/l (12.0%, *p* = 0.005) and CRP by 1.0 mg/l (37.0%, *p* = 0.032) were observed. These data suggest that SBS-LE may have potential as an add-on preventive strategy for residual cardiovascular risk reduction.

## 1. Introduction

Residual cardiovascular risk remains an important driver of atherosclerotic cardiovascular disease progression and adverse events, with triglycerides and inflammation considered important components of such risk [1]. Regarding omega-3 or n-3 fatty acids, high-dose icosapent ethyl (2 g, twice daily) should be considered in combination with a statin in high-risk or very-high-risk patients with elevated triglyceride levels—as recommended by the 2025 Focused Update of the European Society of Cardiology/European Atherosclerosis Society Guidelines for the management of dyslipidemias—to reduce the risk of cardiovascular events [2]. In the REDUCE-IT trial, the addition of high-dose EPA–icosapent ethyl resulted in a significantly lower ischemic cardiovascular event rate among patients with elevated triglyceride levels under the background of statin therapy [3]. In contrast, the STRENGTH trial did not demonstrate a major reduction in adverse cardiovascular events associated with the administration of EPA and DHA in statin-treated patients. Therefore, the clinical efficacy of omega-3 fatty acids remains unclear.

Plant-derived extracts have the potential to serve as beneficial agents for residual risk reduction. Sea buckthorn (*Hippophae rhamnoides* L.) is a multipurpose plant, containing different valuable bioactive substances, including antioxidants, vitamins, fiber, organic acids, proteins, amino acids, mineral elements and lipids with an advantageous and balanced fatty acid composition [4,5]. Pulp, seeds or whole berries of sea buckthorn can be processed to make oil [6]. The berry oil of sea buckthorn is rich in monounsaturated omega-7 fatty acids (palmitoleic acid, hexadecatrienoic acid, heptadecenoic acid, and vaccenic acid), omega-9 fatty acid (oleic acid), and polyunsaturated omega-6 (linoleic acid) and omega-3 (alpha-linolenic acid) fatty acids [7,8]. Sea buckthorn seed oil has been shown to contain a higher level of polyunsaturated fatty acids when compared to whole fruits and pulp [9]. Plant-derived α-linolenic acid can be converted into EPA and DHA in the human body [10]. Animal studies have revealed sea buckthorn seed oil to be effective in reducing cholesterol levels in hamsters [11] and rabbits, as well as lowering triglyceride levels and conferring protection against oxidative stress [12]. Additionally, the anti-inflammatory properties of this nutraceutical have been described, including downregulation of the nuclear factor κB (NF-κB) pathway [13], which could also contribute non-lipid-related cardiovascular benefits.

Data regarding the effects of sea buckthorn-derived lipid extracts on plasma lipid profiles and cardiovascular risk reduction in humans remain limited. Additionally, most available data have been obtained through studies focused on isolated fatty acids instead of those extracted from sea buckthorn oil, thus missing the opportunity to detect their synergistic effects [14].

The aim of this study was to evaluate the effects of a sea buckthorn seed lipid extract (SBS-LE) as an add-on therapy to help reduce residual cardiovascular risk in high-risk patients. Our hypothesis was that the SBS-LE formulation has favorable effects on residual cardiovascular risk parameters in these patients.

## 2. Materials and Methods

### 2.1. Study Design and Participants

This pilot observational study was conducted at the Latvian Centre of Cardiology at Pauls Stradins Clinical University Hospital, Riga, Latvia. Eligible participants were aged 40–70 years with a diagnosis of chronic coronary syndrome. Patients were enrolled if they were on stable (for at least 2 months) maximally tolerated statin therapy (atorvastatin or rosuvastatin), with or without ezetimibe, but had not achieved their guideline-recommended target blood levels for cholesterol. The main exclusion criteria were recent acute myocardial infarction, a planned coronary intervention or surgery, severe heart failure, active severe liver disease and known hypersensitivity to any of the product’s components. All participants signed a written informed consent form. The study protocol was approved by the Ethics Committee of the University of Latvia (meeting protocol Nr. 2; 26 May 2021), and the study was conducted in accordance with the Declaration of Helsinki.

### 2.2. Intervention and Procedures

Enrolled patients were prescribed SBS-LE, a commercially available food supplement (Registry of Food Supplements of Latvia No. 13869) provided by SIBIOTECH (Riga, Latvia). The product was prescribed at a dose of 1000 mg in soft gelatin capsules, taken twice daily (total 2 g/day) for three months in addition to standard medical treatment. This dose was selected as it is a standard, commercially available formulation and is well within the safe range established in the previous literature, while being substantial enough to potentially observe biological effects [15].

At baseline, data regarding medical history and concomitant medications were gathered: body mass index (BMI), blood pressure, and heart rate were measured, while blood samples were taken for determination of plasma lipid profile (total cholesterol (TC), low-density lipoprotein cholesterol (LDL-C), high-density lipoprotein cholesterol (HDL-C), triglycerides), fasting plasma glucose, glycated hemoglobin (HbA1c), C-reactive protein (CRP) and uric acid. All standard clinical laboratory tests were performed at a leading certified national laboratory (E. Gulbja laboratorija) using their standard, automated methods.

After one month, a telephone interview was conducted, and patients were questioned about possible side effects and adverse events. At the three-month follow-up visit, data on adverse events and concomitant medications were acquired, and the same clinical parameter and blood test evaluations as at baseline were performed. Adverse event endpoints were defined as cardiovascular or cerebrovascular events or hospitalization.

### 2.3. Bioavailability Sub-Study

Additionally, the bioavailability of the medication was tested in five volunteers. Blood samples were drawn at the hospital before medication use and at four time points thereafter: 1 h, 6 h, 25 h and 31 h. Samples were analyzed for fatty acid profiles at the Institute “BIOR”. Given the intensive sampling protocol, a small cohort of five volunteers was used for this preliminary, exploratory analysis to confirm the absorption of fatty acids. The amounts of different fatty acids are expressed as relative percentages of total fatty acids in the sample, as parallel triglyceride analysis was not performed.

### 2.4. Product Manufacturing and Composition

Sea buckthorn seed oil was produced from the purified seed of sea buckthorn berries. The seeds were ground to a 1 mm size and dried to a moisture content below 3%. The conditioned sea buckthorn seeds were then extracted via supercritical fluid extraction with carbon dioxide for 3 h, at a pressure of 300 bar and temperature below 50 °C. This process yielded a whole-seed lipid extract, not a molecular distillate or a mixture of isolated fatty acids. The extraction ratio of seed to oil is 10:1.

The extraction of bioactive compounds from sea buckthorn seeds was performed using a modern and environmentally friendly extraction technology, namely, supercritical fluid extraction with carbon dioxide (SC-CO_2_). CO_2_ is recognized as a safe and harmless solvent, and the SC-CO_2_ extraction technology is considered to be efficient and gentle, ensuring that all active nutrients are preserved and concentrated in the final product. This technology does not use organic solvents, and the resulting extract is pure in its natural chemical form. There is no risk of oxidation as the extraction environment is oxygen-free. Extracts do not contain inorganic salts, proteins, fiber, or allergens, as well as heavy metals and other contaminants. This extraction technology is considered solvent-free.

To determine the precise composition of the resulting whole-seed extract, the oil was chemically characterized. The quantification of fatty acids and beta-sitosterol was performed at the Institute “BIOR” using validated in-house gas chromatographic techniques with flame ionization detection (GC-FID). The fatty acid analysis was harmonized with ISO 12966 standards: lipids were extracted (hexane–acetone mixture), transesterified to fatty acid methyl esters (FAMEs) with sodium methoxide, separated on a highly polar column (RT-2560), and quantified against a certified FAME reference standard. Beta-sitosterol was quantified using betulin as an internal standard. The unsaponifiable fraction was extracted using chloroform after alkaline saponification, purified via solid-phase extraction, derivatized to volatile silyl ethers, and analyzed via GC-FID [16].

The final product was formulated in the form of soft-shell capsules. Each capsule contained 1000 mg of the complete SBS-LE. Based on the chemical characterization, each 1000 mg dose of the whole oil was confirmed to naturally provide approximately 300 mg of omega-3 alpha-linolenic acid, 370 mg of omega-6 linoleic acid, 170 mg of omega-9 oleic acid and 7 mg of plant sterol beta-sitosterol. To minimize the potential batch-to-batch variability inherent in natural product formulations, a single standardized production batch of SBS-LE capsules was used for all participants throughout the duration of this study.

### 2.5. Statistical Analysis

Data analysis was carried out with the IBM SPSS Statistics 23.0 software. Central tendency measures for continuous variables are presented as the mean and standard deviation (±SD). Baseline and follow-up changes in the parameters of interest were evaluated with the Wilcoxon test for non-parametric data and paired samples T-test for normally distributed data. The level of statistical significance was set at α = 0.05.

## 3. Results

Prior to the administration of the study product, gas chromatography with flame ionisation detection (GC-FID) analysis confirmed the composition of the specific production batch. The 1000 mg soft gel capsules were verified to contain approximately 300 mg of omega-3 alpha-linolenic acid (ALA), 370 mg of omega-6 linoleic acid (LA), 170 mg of omega-9 oleic acid (OA), and 7 mg of beta-sitosterol.

### 3.1. Patient Population and Hypolipidemic Therapy

A total of 86 patients were enrolled in the trial. Their baseline characteristics are summarized in Table 1.

A one-month telephone follow-up was completed by all participants. A full three-month follow-up was completed by 66 patients; the other 20 patients refused the clinical onsite visit and drawing of blood. Nevertheless, a telephone interview was conducted with these participants to gather data on their vital status and possible adverse events.

All study participants were taking a statin with or without ezetimibe. A majority (77.9%, N = 67) of study patients were on high-intensity statin treatment (atorvastatin 40 and 80 mg or rosuvastatin 20 and 40 mg). Data regarding the use of hypolipidemic therapies are summarized in Table 2. Among the 66 patients who completed the three-month follow-up, only 2 (3.0%) reported changes in lipid-lowering therapy (ezetimibe discontinuation). There were no changes in statin therapy throughout the study period.

### 3.2. Clinical Outcomes

The results regarding the effects of the SBS-LE product on blood pressure and heart rate parameters are summarized in Table 3. A significant reduction in systolic blood pressure by 2.9 mmHg (2.1%) after three months was observed.

### 3.3. Effect on Laboratory Results

**Figure 1:**
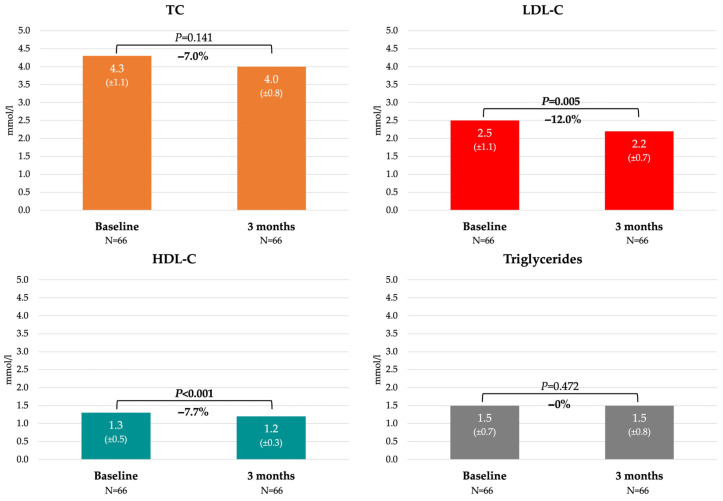
Lipid profile changes.

The changes in lipid parameters with respect to the baseline and three-month results are summarized in Figure 1. A significant reduction in LDL-C by 0.3 mmol/l (12.0%) was established, alongside a decrease in HDL-C and no significant impact on TC or triglycerides after 3 months.

A comparison of the other blood test results is presented in Table 4. A significant reduction in CRP by 1.0 mg/l (37.0%) was determined.

### 3.4. Medication Tolerability and Adverse Events

Overall, good tolerability of the medication was reported. Three patients (3.5%) reported gastrointestinal problems—nausea, dyspeptic complaints and altered bowel movements—which were the most common side effect. This was also the reason for discontinuation of the medication in these study participants. One patient (1.2%) had difficulties swallowing the capsules, leading to medication discontinuation, while another patient (1.2%) reported hemorrhoidal bleeding one month after medication initiation, also leading to discontinuation. Three patients (3.5%) discontinued use of the medication without any objective reason.

At the one-month follow-up, one patient (1.2%) experienced hospitalization due to worsening heart failure. At the time of the three-month follow-up, one patient (1.2%) had undergone scheduled coronary angiography with immediate percutaneous coronary intervention with a drug-eluting stent implantation for a critical stenosis in the left anterior descending artery.

### 3.5. Bioavailability

**Figure 2:**
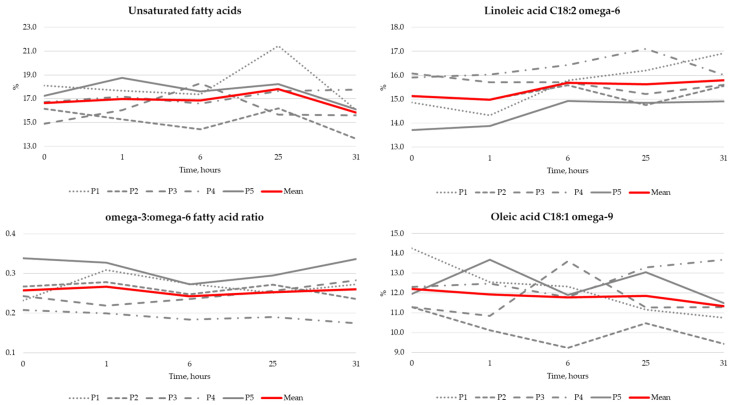
Different fatty acid concentration changes in blood samples after SBS-LE intake.

The bioavailability of SBS-LE was determined in the five volunteers enrolled in the sub-study. The results regarding the time-related concentrations of different fatty acid types in blood samples taken after SBS-LE use are summarized in Figure 2. EPA levels were undetectable (<0.1) in almost all samples (22 out of 25 total, 88.0%), gamma-linolenic omega-6 fatty acid was undetectable in 23 of 25 samples (92.0%), and alpha-linoleic acid omega-3 fatty acid was also undetectable in majority of samples (15 of 25, 60.0%), with all five bioavailability readouts available only for one patient. Therefore, these data are not graphically represented.

## 4. Discussion

In this pilot study, the main findings were reductions in systolic blood pressure, LDL-C and CRP levels at the three-month follow-up with the addition of an SBS-LE formulation to optimal medical therapy in chronic coronary syndrome patients.

After three months of use, there was a significant difference in systolic blood pressure with the use of the SBS-LE formulation, being 2.9 mmHg (2.1%) lower in the patients receiving SBS-LE. An animal study has demonstrated that supplementation with sea buckthorn seed oil resulted in blood pressure normalization after 30 days, with reductions in both systolic and diastolic blood pressure (by 9.57 mmHg and 4.96 mmHg, respectively) [15]. The antihypertensive properties of sea buckthorn have been attributed to flavones, and the suggested mechanisms underlying its antihypertensive activity include protection of endothelial function, reduction in reactive oxygen species and inhibition of blood platelet aggregation [5]. The effects of flavonoids present in sea buckthorn have also been described as comparable to those of calcium channel blockers and angiotensin-converting enzyme inhibitors in reducing diastolic blood pressure [17]. Nevertheless, when comparing the findings, it is important to emphasize the differences between study subjects, as well as the fact that data available from recent human studies remains limited.

Regarding the effects on plasma lipid profiles, in our study, there was a significant reduction in LDL-C by 0.3 mmol/L (12.0%) without a significant effect on total cholesterol, as well as a decrease in HDL-C. The positive properties of sea buckthorn oil-derived fatty acids on lipid profiles, including lowering of LDL-C, have been established; however, their effects on HDL-C have differed in studies with separate fatty acid implementations. Nevertheless, the importance of sea buckthorn oil intake with respect to its bioactive components has been emphasized [14]. In a study involving hamsters, sea buckthorn seed oil reduced the total cholesterol level by 20–22%. The mechanism proposed in this research was increased intestinal cholesterol excretion and modulation of the gut microbiota by promoting the growth of short-chain fatty acid-producing bacteria [11]. An in vivo study involving rats demonstrated significant reductions in both LDL-C and HDL-C, similar to the results reported here [18]. It has also been proposed that sea buckthorn does not affect normal lipid levels but lowers excess cholesterol [19].

Inflammation is an important driver of residual cardiovascular risk [20]. The anti-inflammatory properties of sea buckthorn oil have been described, associated with a reduction in the release of various proinflammatory factors. Active substances in sea buckthorn have been shown to inhibit lipopolysaccharide-mediated processes, block the NF-κB and mitogen-activated protein kinase (MAPK) signaling pathways, reduce the levels of inflammatory cytokines, and regulate the expression of the adhesion molecules intercellular adhesion molecule 1 (ICAM-1) and vascular cell adhesion molecule 1 (VCAM-1) [17,21]. A study involving rats demonstrated the anti-inflammatory effects of a sea buckthorn flavone, which were attributed to an increase in complement-inhibiting C1q/tumor necrosis factor (TNF)-related protein 6 (CTRP6) secretion in peripheral blood macrophage cells, resulting in a reduction in inflammatory cytokine levels. Additionally, the suppression of cell foaming was observed [22]. Another study involving rats demonstrated that polyphenols isolated from sea buckthorn berries decreased the expression levels of serum TNF-α and interleukin-6 [23]. In our study, a reduction in CRP levels by 1.0 mg/l (37.0%) was established, representing the potential suppression of inflammatory processes. A clinical study involving women who consumed sea buckthorn juice also demonstrated a significant reduction in CRP [24].

Additional bioavailability testing was performed in this study, allowing the concentrations of different fatty acids in blood samples to be analyzed. Although there were no significant changes in the analyzed fatty acids, the overall most prominent tendency observed was a slight increase in omega-6 fatty acid levels. It is also important to highlight that the presented bioavailability lipidomics data are exclusive to the specific SBS-LE formulation that was produced for this clinical trial. Regarding the fatty acid profile, it is important to emphasize that the primary omega-3 in this plant-based extract is ALA. The absence of marine-derived fatty acids, such as EPA and DHA, is the correct and expected chemical profile for this product.

This study has several important limitations. Primarily, it is a single-arm, observational pilot study without a comparator or placebo group. Therefore, we cannot definitively attribute the observed changes solely to the SBS-LE intervention. The influence of the placebo effect, the natural course of the disease, or other unmeasured confounding variables cannot be ruled out. Furthermore, the small sample size (n = 86) and the significant loss to follow-up (20 patients) reduce the statistical power and generalizability of our findings. Additionally, the short follow-up period of three months does not allow for the assessment of longer-term outcomes. Therefore, these findings should be interpreted as preliminary and hypothesis-generating. A future, large-scale, double-blind randomized controlled trial will be necessary to confirm these potential effects.

## 5. Conclusions

The results of this exploratory pilot study suggest that the use of omega-3, -6, and -9 fatty acids bioactive compound-containing SBS-LE, produced via supercritical fluid extraction with carbon dioxide, was associated with statistically significant reductions in systolic blood pressure, LDL-C and CRP levels after 3 months. These preliminary findings indicate that SBS-LE warrants further investigation as a potential add-on therapy to standard treatment for the reduction in residual cardiovascular risk.

## Figures and Tables

**Table 1:** Patient baseline characteristics.

Total Number of Patients		N = 86
Sex	Male	54.7% (N = 47)
	Female	45.3% (N = 39)
Age, years		73.2 (±9.9)
BMI, kg/m^2^		28.7 (±5.0)
Waist circumference, cm		102.8 (±15.6)
Dyslipidemia		100.0% (N = 86)
Systolic blood pressure, mmHg		136.4 (±12.8)
Diastolic blood pressure, mmHg		78.9 (±6.4)
Heart rate, x/min		70.2 (±8.4)
TC, mmol/L		4.4 (±1.0)
LDL-C, mmol/L		2.7 (±1.1)
HDL-C, mmol/L		1.3 (±0.5)
Triglycerides, mmol/L		1.5 (±0.7)
Fasting plasma glucose, mmol/L		6.2 (±1.7)
HbA1c, %		6.3 (±1.0)
CRP, mg/L		3.7 (±3.8)
Uric acid, umol/L		368.3 (±89.0)

**Table 2:** Hypolipidemic therapy.

Baseline N = 86	Atorvastatin	31.4% (N = 27)
	High-intensity atorvastatin *	19.8% (N = 17)
	Rosuvastatin	68.6% (N = 59)
	High-intensity rosuvastatin **	58.1% (N = 50)
	Ezetimibe	59.3% (N = 51)
3-month follow-up N = 66	Statin intensification	0% (N = 0)
	Statin de-escalation	0% (N = 0)
	Addition of ezetimibe	0% (N = 0)
	Ezetimibe discontinuation	3.0% (N = 2)

* Atorvastatin 40 and 80 mg. ** Rosuvastatin 20 and 40 mg.

**Table 3:** Changes in blood pressure and heart rate parameters.

	Baseline N = 66	3-Month Follow-Up N = 66	*p*
Systolic blood pressure, mmHg	137.7 (±12.9)	134.8 (±12.7)	0.012
Diastolic blood pressure, mmHg	79.2 (±6.5)	78.9 (±7.0)	0.585
Heart rate, x/min	70.6 (±9.0)	69.0 (±7.5)	0.061

**Table 4:** Changes in laboratory parameters.

	Baseline N = 66	3-Month Follow-Up N = 66	*p*
Fasting plasma glucose, mmol/L	6.2 (±1.6)	5.9 (±1.5)	0.242
HbA1c, %	6.4 (±0.9)	6.6 (±1.2)	0.153
CRP, mg/L	2.7 (±2.6)	1.7 (±1.6)	0.032
Uric acid, umol/L *	364.0 (±78.5)	375.9 (±79.5)	0.105 *

* Normally distributed data, analyzed via parametric test.

## Data Availability

Data generated or analyzed in this study that support the findings are included in the article. Further enquiries can be directed to the corresponding author upon reasonable request.

## Abbreviations

The following abbreviations are used in this manuscript:

BMI	Body mass index
CRP	C-reactive protein
CTRP6	C1q/TNF-related protein 6
DHA	Docosahexaenoic acid
EPA	Eicosapentaenoic acid
HbA1c	Glycated hemoglobin
HDL-C	High-density lipoprotein cholesterol
ICAM-1	Intercellular adhesion molecule 1
IQR	Interquartile range
LDL-C	Low-density lipoprotein cholesterol
MAPK	Mitogen-activated protein kinase
NF-κB	Nuclear factor κB
SBS-LE	Sea buckthorn seed lipid extract
SC-CO2	Supercritical fluid extraction with carbon dioxide
SD	Standard deviation
TC	Total cholesterol
TNF	Tumor necrosis factor
VCAM-1	Vascular cell adhesion molecule 1
